# How race, sex and age interact in association with COVID-19 outcomes over time: An analysis of Michigan data

**DOI:** 10.1371/journal.pone.0288383

**Published:** 2023-08-31

**Authors:** Max Jordan Nguemeni Tiako, Alyssa Browne

**Affiliations:** 1 Department of Medicine, Brigham and Women’s Hospital, Boston, MA, United States of America; 2 Harvard Medical School, Boston, MA, United States of America; 3 Department of Sociology, UNC Chapel Hill, Chapel Hill, NC, United States of America; 4 Carolina Population Center, UNC Chapel Hill, Chapel Hill, NC, United States of America; University of California Davis School of Medicine, UNITED STATES

## Abstract

**Background:**

COVID-19 has had a disproportionate impact on racial and ethnic minorities compared to White people. Studies have not sufficiently examined how sex and age interact with race/ethnicity, and potentially shape COVID-19 outcomes. We sought to examine disparities in COVID-19 outcomes by race, sex and age over time, leveraging data from Michigan, the only state whose Department of Health and Human Services (DHSS) publishes cross-sectional race, sex and age data on COVID-19.

**Methods:**

This is an observational study using publicly available COVID-19 data (weekly cases, deaths, and vaccinations) from August 31 2020 to June 9 2021. Outcomes for descriptive analysis were age-standardized COVID-19 incidence and mortality rates, case-fatality rates by race, sex, and age, and within-gender and within-race incidence rate ratios and mortality rate ratios. We used descriptive statistics and linear regressions with age, race, and sex as independent variables.

**Results:**

The within-sex Black-White racial gap in COVID-19 incidence and mortality decreased at a similar rate among men and women but the remained wider among men. As of June 2021, compared to White people, incidence was lower among Asian American and Pacific Islander people by 2644 cases per 100,000 people and higher among Black people by 1464 cases per 100,000 people. Mortality was higher among those aged 60 or greater by 743.6 deaths per 100,000 people vs those 0–39. The interaction between race and age was significant between Black race and age 60 or greater, with an additional 708.5 deaths per 100,000 people vs White people aged 60 or greater. Black people had a higher case fatality rate than White people.

**Conclusion:**

COVID-19 incidence, mortality and vaccination patterns varied over time by race, age and sex. Black-White disparities decreased over time, with a larger effect on Black men, and Older Black people were particularly more vulnerable to COVID-19 in terms of mortality. Considering different individual characteristics such as age may further help elucidate the mechanisms behind racial and gender health disparities.

## Introduction

Racial disparities in U.S. COVID-19 incidence and mortality have been well documented. Three years into the pandemic and across iterations of COVID-19 surges, Black, Hispanic and Native people have most consistently died of COVID-19 at higher rates compared to their White and Asian counterparts [1, 2]. Racial COVID-19 disparities are mediated by a confluence of inequities in occupational and residential risk, as well as pre-existing disparities in health status and access to healthcare. For example, Black and Hispanic people are overrepresented among occupations deemed essential during the pandemic, and less likely to be able to work remotely compared to White counterparts [3–5]. Further, documented racial disparities in COVID-19 mortality remains when adjusting for age [1], yet most analyses have not conducted subgroup analysis of racial disparities in mortality, incidence and vaccination rates by age and sex due to limitations and discrepancies in data sources. It is critical to interrogate subgroup variation by age and sex [2, 6, 7].

In addition to racial and ethnic disparities in COVID-19 outcomes, studies have highlighted gender disparities, mediated by the gendering of certain occupations deemed essential, disproportionately putting women who work in food services, hospitality, and healthcare at increased risk of infection [4, 5]. At the same time, evidence shows that female sex is protective against COVID-19 severity and mortality, conditional on age [8]. One study found that the majority of U.S. states reported greater mortality among male COVID-19 patients [9], and in line with immunological research, the sex mortality gap was smaller among older patients [8]. Recent scholarship disaggregating COVID-19 trends by sex finds that sex disparities in mortality and incidence are inconsistent, varying across time and space and likely dependent on context and characteristics such as race and ethnicity [10].

Few studies have explored the intersection of sex, race/ethnicity, and age and the impact on COVID-19 outcomes over time. To date, only two cross-sectional studies have examined racial and sex disparities in COVID-19 mortality, one in the states of Michigan and Georgia [10] and the other at the national level [11]. Both studies found that sex disparities in mortality were wider among racial and ethnic minority groups than White people [11]. Also emphasized was the greater mortality burden among women of color compared to White men. These disparities are likely attributable to the aforementioned material conditions that disproportionately affect communities of color. In November 2020, the Black mortality rate by COVID-19 in Michigan was more than 35%. As of April 15, 2022, Black individuals make up more than 17% of deaths despite comprising approximately 14.1% of the population [12, 13]. This suggests that mortality disparities may be improving over time, yet the research is sparse on whether these improvements in the disparity are being driven by a particular age or gender strata within Black individuals. Prior research examining race and age-stratified COVID-19 mortality in Michigan focused on a shorter period (March through October 2020) and found that among 6,065 COVID-19 related deaths, the mortality rate for Black individuals was 3.6 times that of White individuals. Further, Black individuals across all intersections of age, race, sex, and prior comorbidities were at higher risk of COVID-19 mortality than their White counterparts [14].

Mitigation efforts have evolved throughout the pandemic, with varying uptake and access across different groups. Indeed, vaccination coverage among U.S. adults was lower among those living in counties with lower socioeconomic status as of summer 2021 [15]. Additionally, until recently, Black and Hispanic people were less likely to receive COVID-19 vaccinations than White people [16]. Despite vaccination efforts, the state of Michigan faced a significant surge in COVID-19 cases and mortality during the spring of 2021 compared to other states, disproportionately affecting younger people, in part due to the age prioritization schema in vaccine distribution [17].

This paper expands on previous cross-sectional analysis by Rushovich et al. [10] and Xu et al. [11] by examining COVID-19 disparities in age-standardized incidence, mortality, and vaccination rates by race and sex over time. Leveraging publicly available weekly data from the Michigan Department of Health and Human Services, we examine trends in COVID-19 incidence, mortality, and vaccination coverage over 38 weeks (August 31 2020—June 9 2021); we focused on Michigan as the only state that publishes cross-sectional COVID-19 data that includes race, age and sex. Given the dynamic nature of the COVID-19 pandemic and of mitigation efforts, examining subgroup trends over time offers a deeper understanding of how to better mitigate disparities.

## Materials and methods

### Study type & data sources

This is an observational study using publicly available data from the Michigan Department of Health and Human Services (HHS). HHS reports weekly cases, deaths, and vaccinations by age group, sex, and race/ethnicity. Using the Internet Archive’s “Wayback Machine,” we retroactively downloaded Michigan COVID data for one date per week beginning August 31, 2020, until June 9, 2021, resulting in a longitudinal dataset that spans 38 weeks. Additionally, we relied on the published vaccination prioritization schedule for Michigan to identify vaccine eligibility by age [18]. We used the 2019 bridged-race population estimates from the Centers for Disease Control and Prevention [19] as population denominators in all rate calculations reported per 100,000 people. Lastly, we used the U.S. standard million from 2000 to age-standardized race-gender specific rates to allow for comparison across groups. Because we used publicly available, ecological data with no individual human subject information, this study was deemed exempt from review by the Yale University IRB.

## Outcomes and covariates

Outcomes of our study were the following: for descriptive time trends, weekly cumulative age-standardized COVID-19 incidence and mortality rates, and within-sex and within-race incidence rate ratios (IRR) and mortality rate ratios (MRR); and for linear regression, age-specific COVID-19 incidence rate, mortality rate, and case-fatality rate by race and sex.

Available data on race is reported using the following categories: American Indian/Alaska Native, Asian/Pacific Islander, Black/African American, multiple races, other, refused, unknown, and White. For this analysis, we dropped the categories “multiple races,” “other,” “refused,” and “unknown” because we could not match these data to the 2018 bridged-race population estimates from CDC that we used as population denominators for each age-race-sex group in all calculations. No information was provided by Michigan HHS on how race data was collected. Sex was reported by Michigan HHS as male and female, with no information provided on whether this is self-report. Age groupings are 0–39, 40–59, and 60+ to harmonize age groups across data sources. We created indicators to denote when specific age groups were vaccine eligible.

### Statistical analysis

We first generated age-specific incidence and mortality rates and age-specific case fatality ratios (CFR) for each age-race-sex group. Age-standardized rates and ratios (incidence, mortality, and case fatality) were then calculated by multiplying age-standardized rates and ratios by the standard population distribution. We then calculated within-sex and within-race incidence rate ratios and mortality rate ratios to demonstrate racial and gender disparities in COVID incidence and mortality over time.

**Fig 1:**
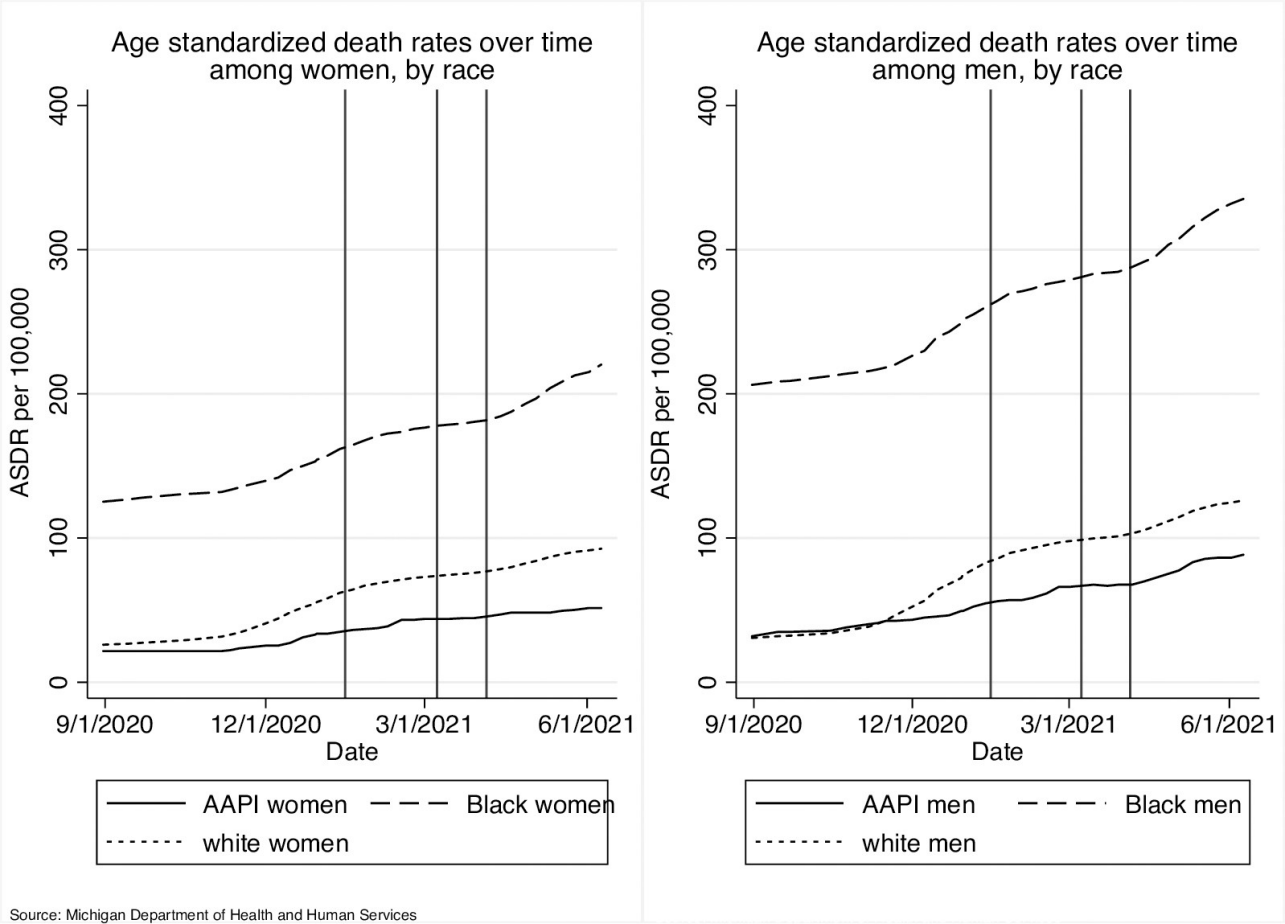
Time trends in age standardized incidence and mortality rates by race and sex (Vertical reference lines mark the dates that the state of Michigan expanded vaccine eligibility).

**Fig 2:**
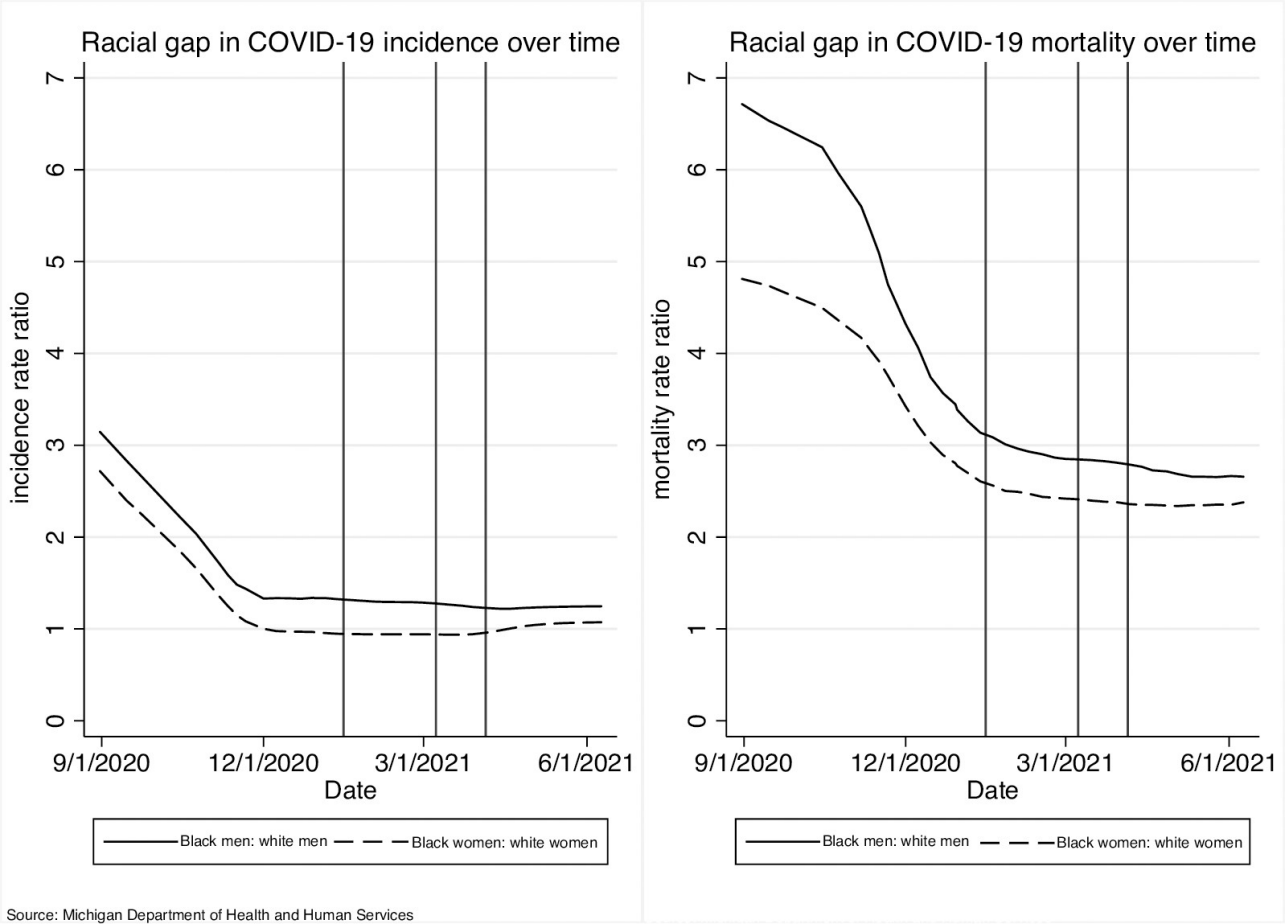
Black-White racial gaps in COVID-19 incidence and mortality, by sex.

**Fig 3:**
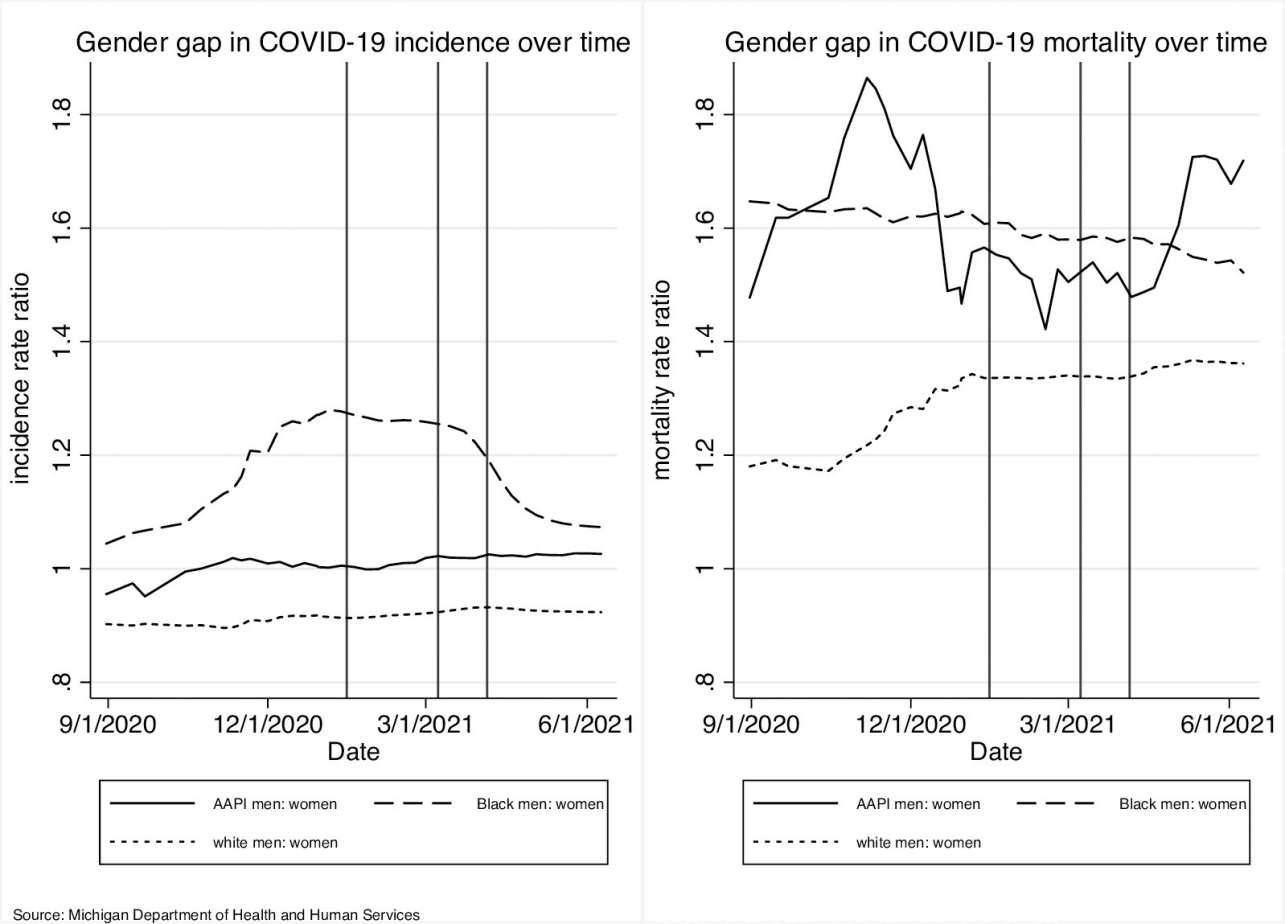
Within-race sex disparities in COVID-19 incidence and mortality. **Note:** Figs 1–3 draw on administrative data capturing the full population and plot age-standardized rates and ratios. While there are mixed opinions regarding the incorporation of confidence intervals in the use of population data, age standardized rates should not be compared by assessing overlapping of confidence intervals, per the Washington State Department of Health “Guidelines for Using Confidence Intervals for Public Health Assessment.” As such, Figs 1–3 do not include confidence intervals.

We plotted age-standardized rates by race-sex groups to descriptively visualize the time trends in COVID-19 cumulative incidence and mortality (Fig 1), and within-race and within-sex incidence rate ratios and mortality rate ratios to descriptively visualize disparities in COVID-19 outcomes over time (Figs 2 and 3). This allows us to compare trends more broadly by race-sex groups and visualize temporal trends in COVID-19 disparities by sex and race. We then conducted linear regressions with age-specific incidence rates, age-specific mortality rates, and age-specific case fatality ratios as dependent variables, and race, age, and sex as independent variables. We also conducted these regressions with interaction terms between age and race, race and sex, and age and sex.

## Results

### Trends in incidence, mortality, and vaccination coverage, by race and sex

Fig 1 plots time trends in age standardized incidence rates (top panel) and mortality rates (bottom panel) by race and sex. As cumulative incidence and mortality rates climbed for all race-sex groups over the study period, the rate of change among AAPI women and AAPI men was less steep than for their White and Black counterparts. In fact, incidence and mortality remained consistently lower for AAPI women relative to Black and White women and for AAPI men relative to Black and White men, with the gap in incidence and mortality widening over time.

To examine Black-White racial gaps in age-standardized incidence and mortality more closely, we plotted within-sex Black:White incidence rate ratios and mortality rate ratios (Fig 2). We noted that within-sex racial disparities in COVID-19 incidence and mortality declined over time. The Black-White racial gap in incidence decreased at a similar rate among men and women over time, dropping nearly to 1 for both men and women, although the gap remained slightly higher among men (1.24 vs. 1.07). In terms of COVID-19 mortality, we observed a steeper drop in the within-sex Black-White mortality gap among men over time, bringing the mortality disparity between Black men and White men to a similar, although still slightly higher, level as women (2.65 vs 2.38).

To more closely examine male-female gaps in age-standardized incidence and mortality, we plotted within-race sex disparities (see Fig 3). We found that, over the observed period, the male:female incidence rate ratio remained greater than 1 among Black men and women, below 1 among White men and women, and close to 1 among AAPI men and women. While within-race male:female incidence rate ratios remained relatively steady throughout the study period among White and AAPI men and women, we found an initial increase in the relative incidence among Black men compared to Black women, which plateaued around the same time as Michigan began its vaccination rollout for healthcare workers (as indicated by the first vertical bar, representing December 14^th^ 2020), and further decreased as vaccines became more widely available to the broader population (March 1^st^ 2021, available for all essential workers; April 5^th^ 2021, available for the entire population) [18].

The male:female mortality rate ratio remained steadily above 1 for all racial groups, indicating that men consistently experienced higher rates of COVID-19 mortality compared to women from the same racial group over time. The mortality disparity declined slightly over time among Black men and women. Among White men and women, the male:female mortality rate ratio was consistently lower compared to Black men and women, but steadily widened during the study period.

### Regression results

Beyond describing race and gender trends in COVID-19 incidence and mortality, and racial and gender disparities in COVID-19 outcomes over time, we sought to examine how race, sex, and age were associated with COVID-19 outcomes. To achieve this aim, we used data from our last observed week (the week of June 9, 2021) and linear regression to examine the relationship between race, sex, and age group with COVID-19 incidence, mortality, and case fatality. Table 1 describes incidence, mortality, case fatality, and vaccination rates as of June 9, 2021, by racial group, age category, and sex.

**Table 1:** COVID-19 incidence, mortality, and vaccination rates by race, age, and sex, Michigan, June 2021.

Group, by age, race and gender	Deaths per 100,000 people	Incidence per 100,000 people	% of those infected with COVID who died (CFR)	% of each race-sex group who are fully vaccinated
AAPI man 0–39	0	3562.9	-	43.4
Black man 0–39	15.8	6311.1	0.3	24.3
White man 0–39	4.4	5961.1	0.1	36.9
AAPI woman 0–39	0	3453.7	-	42.9
Black woman 0–39	11.1	6796.7	0.2	26.9
White woman 0–39	2.9	6824.7	0.04	41.2
AAPI man 40–59	15.4	3937.2	0.4	43.4
Black man 40–59	283.5	10143.2	2.8	24.3
White man 40–59	65.6	6865.9	1.0	36.9
AAPI woman 40–59	14.4	4084.2	0.4	42.9
Black woman 40–59	155.1	8173.7	1.9	26.9
White woman 40–59	39.7	7181	0.6	41.2
AAPI man 60+	510.7	3717.9	13.7	43.4
Black man 60+	1520.1	8481.7	17.9	24.3
White man 60+	642.9	5736.2	11.2	36.9
AAPI woman 60+	288.5	3292.8	8.8	42.9
Black woman 60+	1046.5	6791.2	15.4	26.9
White woman 60+	487.1	5344	9.1	41.2

Table 2 displays the results from multivariable OLS analyses of COVID-19 incidence and mortality by race and sex. Compared to White people, age and sex-adjusted incidence was lower among AAPI people by 2,644 cases (95% CI -3619.4; -1668.7, P<0.001) and higher among Black people by 1,464 cases (95% CI 488.8; 2439.5, P = 0.01). Adjusting for race and sex, there were 1,246 more cases (270.5; 2221.2, P = 0.02) among people aged 40–59 compared to people aged 40 or younger.

**Table 2:** Results from OLS analyses on COVID-19 incidence, mortality, and case fatality.

IV\DV	Incidence	Mortality	Mortality, accounting for incidence	Case Fatality Ratio
**Sex **				
Male	ref	ref	ref	ref
Female	-308.4 (-1104.7; 488.0)	-112.6 (-337.2; 112.0)	-74.1 (-292.9; 144.6)	-1.2 (-3; 0.6)
**Race **				
White	ref	ref	ref	ref
AAPI	**-2644.1 (-3619.4; -1668.7)** ***	-68.9 (-344.0; 206.1)	260.7 (-253.9; 775.3)	0.2 (-2; 2.4)
Black	**1464.1 (488.8; 2439.5** )**	**298.3 (23.2; 573.3)** *	115.7 (-242.3; 473.8)	**2.8 (0.6; 4.9)** *
**Age **				
<40	ref	ref	ref	ref
40–59	**1245.8 (270.5; 2221.2)** *	89.9 (-185.2; 365.0)	-65.4 (-399.3; 268.5)	1.1 (-1.1; 3.3)
60+	75.6 (-899.7; 1050.9)	**743.6 (468.5; 1018.7)** ***	**734.2 (473.5; 994.8)** ***	**12.6 (10.4; 14.8)** ***
**Incidence **	-	-	0.12 (-0.04; 0.3)	-

*p<0.05

**p< 0.01

***p<0.001

Compared to those aged 0–39, the mortality rate was higher among those aged 60 or greater by 743.6 deaths per 100,000 people (95% CI 468.5; 1018.7, P<0.001). Accounting for incidence, only age remained independently associated with greater mortality: compared to people 40 years old or younger, age 60 or older was associated with 734.2 more deaths (95% CI 473.5, 994.8, P<0.001) per 100,000 people.

In terms of COVID-19 case fatality ratios (CFR), Black people had a greater age and sex-adjusted CFR compared to White people (2.8, 9.5% CI 0.6, 4.9, P = 0.02). Similarly, age 60 or older was independently associated with a greater CFR (12.6, 95% CI 10.4, 14.8, P<0.001) compared to age 40 or younger.

**Fig 4:**
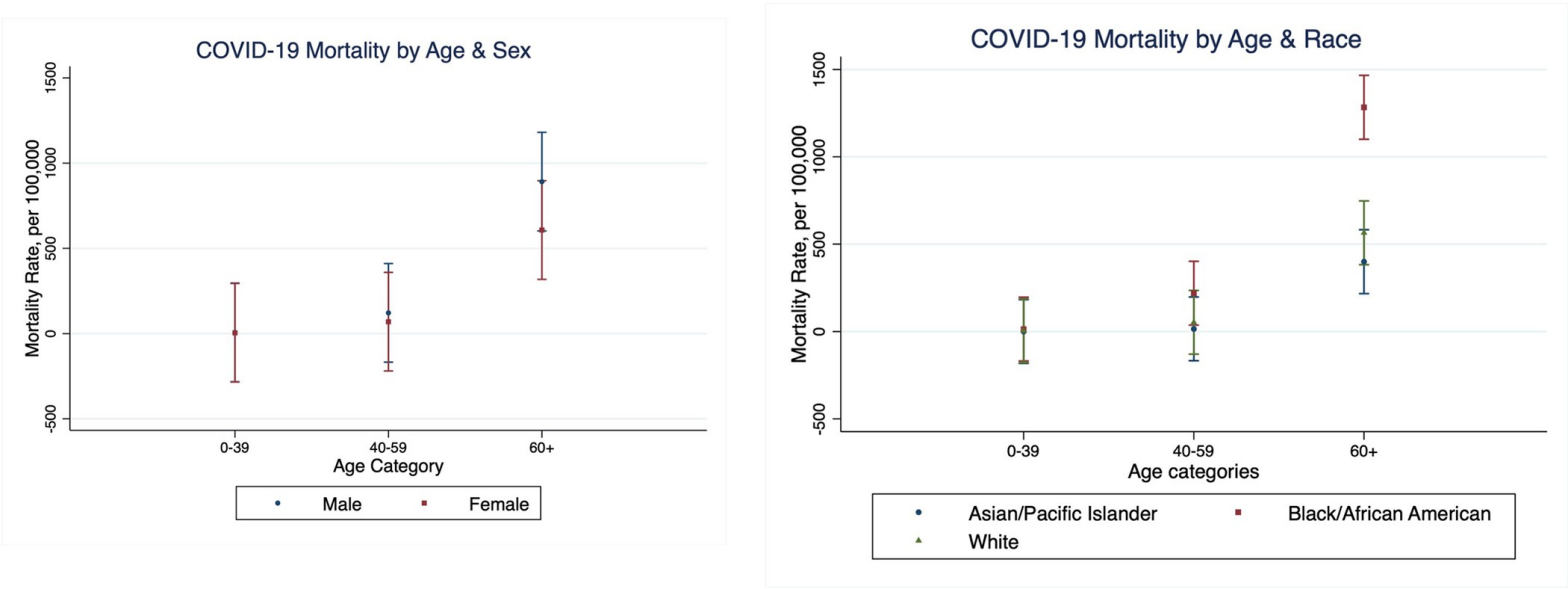
Associations between age & sex and age & race in COVID-19 mortality.

In our multivariable OLS analyses with interaction terms between independent variables (Table 3), the gender gaps in incidence and mortality were wider among Black people compared to other racial groups, but not statistically significant. We found a significant association between age and race: mortality was significantly higher among Black people aged 60 or more compared to AAPI and White people of the same age (interaction term b = 708.5 per 100,000 people, 95% CI 342.9–1074.1, P<0.002), while there was no statistically significant difference in the racial mortality gap in other age groups (Fig 4).

**Table 3:** Summary of regression results.

	Mortality	Mortality, with Race-Age Interaction	Mortality with race-sex interaction	Incidence	Mortality, accounting for incidence	Case Fatality Ratio
Sex						
Male	ref	ref	Ref	ref	Ref	Ref
Female	-112.6 (-337.2; 112.0)	-112.6 (-234.44; 9.27)	-61.1 (-489.9; 367.7)	-308.4 (-1104.7; 488.0)	-74.1 (-292.9; 144.6)	-1.2 (-3; 0.6)
Race						
White	ref	ref	ref	ref	ref	
Asian	-68.9 (-344.0; 206.1)	-3.7 (-262.15; 254.84)	-62.26 (-491.1; 366.6)	-2644.1 (-3619.4; -1668.7)***	260.7 (-253.9; 775.3)	0.2 (-2; 2.4)
Black	298.3 (23.2; 573.3)*	9.8 (-248.68; 268.31)	368.8 (-60; 797.6)	1464.1 (488.8; 2439.5)**	115.7 (-242.3; 473.8)	2.8 (0.6; 4.9)*
Age						
<40	ref	ref			ref	
40–59	89.9 (-185.2; 365.0)	49 (-209.49; 307.49)	89.9 (-213.3; 393.1)	1245.8 (270.5; 2221.2)*	-65.4 (-399.3; 268.5)	1.1 (-1.1; 3.3)
60+	743.6 (468.5; 1018.7)***	561.4 (302.87; 819.85)**	743.6 (440.4; 1046.8)***	75.6 (-899.7; 1050.9)	734.2 (473.5; 994.8)***	12.6 (10.4; 14.8)***
Incidence	-			-	0.12 (-0.04; 0.3)	-
Interaction terms						
Race & Age						
Asian-40-59		-34.1 (-399.66; 331.47)				
Asian-60+		-161.7 (-527.3; 203.83)				
Black-40-59		156.8 (-208.75; 522.38)				
Black-60+		708.5 (342.92; 1074.05)**				
						
Race & Sex						
Asian, Female			-13.3 (-619.8; 593.1)			
Black, Female			-141.1 (-747.6; 465.3)			
R-squared	0.81	0.97	0.82	0.89	0.85	0.92

Table legend

* P<0.05

**P<0.01

***P<0.001, mortality and incidence coefficients reported in per 100,000 people

## Discussion

Our manuscript has five key findings. First, the within-sex Black-White racial gap in COVID-19 incidence decreased at the same rate among men and women over time, although the racial gap remained slightly wider among men. Second, we observed an initial increase in the relative incidence among Black men compared to Black women, which plateaued and further decreased in the same period that vaccines became more widely available. Third, in terms of COVID-19 mortality, the Black-White mortality gap narrowed more steeply among men compared to women over time, but the disparity remained slightly higher among men. Fourth, there was a steady decline in the gender gap in mortality among Black people, while there was an overall increase in the gender gap in mortality among White people, potentially tempered by the introduction of vaccines. Finally, regression results showed that the COVID-19 mortality rate was higher among Black people (especially among those 60 years or older), but lower among AAPI people relative to White people, while vaccination rates were lower among Black people and higher among AAPI people compared to White people.

We use intersectionality as an analytical strategy that provides new angles of vision on social phenomena, COVID-19 disparities in incidence, mortality, and vaccination rates. Doing so provides novel insight on subgroup trends that may elucidate how context, such as gendered and racialized occupational and social structures, may shape the trajectory of COVID-19. Our findings are in line with other studies focused on racial and gender disparities in COVID-19 outcomes. A 2021 study found that, in Michigan, Black men had the highest COVID-19 incidence and mortality, and that the gender gap in mortality was most pronounced among Black people [14].

Novel in our findings is the fact that the gender gap between Black men and women in both incidence and mortality reduced over time. This may suggest that the decrease in the gender gap for Black people after April 2021 may be in part attributable to vaccines. While we observed an initial rise in the White mortality gender gap, it notably tempered halfway through the study period, and was stagnant during the second half. Both findings suggest that the spillover effects of vaccine uptake may be more profound on mortality among men (especially Black men), who have higher incidence and lower vaccine uptake compared to women.

The racial and gender disparities we observed are likely attributable to differences in household contexts, occupational exposures, unique settings such as carceral settings, and disparities in access to healthcare. Black people are more likely to be employed in low-wage, high-risk occupations, and live in multigenerational homes, which increases the risk of at-home spread of COVID-19 [6, 20, 21]. Indeed, studies have found significantly high COVID-19 and excess mortality among jobs disproportionately occupied by Black people [3, 22] In addition, incarceration rates are highest among Black men [23], and evidence shows that infection rates in carceral settings have been higher than in community settings [24]. Furthermore, infections in carceral settings increase spread in the surrounding communities [25, 26]. Access to healthcare, such as having a primary care provider as a trusted source of health information is a known predictor of vaccine uptake, however, Black people are the least likely to report having a primary care provider.

The fact that older Black people had even higher mortality rates may be due to increased transmission risk in association with multigenerational homes in the setting of greater incidence, as well as segregation in health services provision, thus affecting the quality of care. Studies have found that skilled nursing facilities (SNF) are segregated by race, and Black patients are more likely to utilize lower-quality SNFs [27]. In the COVID-19 context, SNF quality correlated with staff having access to protective personal equipment, as well as transmission rates [28, 29].

Our findings have implications for the study of population health disparities. Single axis approaches to identifying disparities along racial or gender lines are insufficient to uncover key social dynamics that shape health outcomes. Investigating multidimensional dynamics in health disparities, especially complex public health phenomena such as the COVID-19 pandemic, is essential to understand and tackle the overlapping economic, domestic and health vulnerabilities that impact COVID-19 outcomes and population health at large. Future work and data collection strategies should prioritize intersectionality and encourage policy makers to implement such strategies to mitigate the widening gender disparities related to COVID-19.

## Limitations

Our study has limitations. First, we use state-level ecological data, and not individual-level data, meaning we are not able to draw any causal relationships such as linking vaccinations to risk of incidence and mortality. Second, we used few characteristics in our analysis due to limited publicly available data. Lastly, this is a single-state analysis, thus findings are not inherently generalizable to the rest of the country. Observational data nevertheless serves an important role in generating hypotheses to ultimately identify drivers of health outcomes.

## Conclusions

Using intersectionality as an analytic framework, we identified COVID-19 incidence, mortality and vaccination patterns that varied over time along the lines of race, gender and age. While there were Black-White disparities in incidence and mortality over the course of the study period, they decreased over time, particularly among Black men, potentially attributable to the introduction of vaccinations. Older Black people were especially more vulnerable to COVID-19 in terms of mortality. Considering different individual characteristics such as age may further help elucidate the mechanisms behind racial and gender disparities in population health.

## Supporting information

S1 Raw data (XLSX)
